# Enhancing Literacy and Communicative Skills of Students With Disabilities in Special Schools Through Dialogic Literary Gatherings

**DOI:** 10.3389/fpsyg.2021.662639

**Published:** 2021-04-15

**Authors:** Aitana Fernández-Villardón, Rosa Valls-Carol, Patricia Melgar Alcantud, Itxaso Tellado

**Affiliations:** ^1^Faculty of Psychology and Education, University of Deusto, Bilbao, Spain; ^2^Department of Theory and History of Education, University of Barcelona, Barcelona, Spain; ^3^Department of Pedagogy, University of Girona, Girona, Spain; ^4^Department of Pedagogy, University of Vic, Vic, Spain

**Keywords:** dialogic literary gatherings, dialogic reading, students with disabilities, special schools, literacy, communicative skills

## Abstract

Enhancing the quality of learning opportunities for students with disabilities and the learning level attained is a pending challenge. This challenge is especially relevant in the context of special schools, where the learning possibilities derived from interactions with others is limited. However, providing these students with a sufficient level of instrumental learning, such as literacy, and communicative and reasoning abilities is crucial for their subsequent educational and social opportunities. In this case study we analyse a special school that has implemented Dialogic Literary Gatherings with their students as a means to increase learning interactions within the group around the reading and debate of classical books. Semi-structured interviews were conducted with the school principal and with a teacher of the transition to adult life course, and two focus groups were conducted with teachers–one with primary education teachers and one with secondary education teachers–and two focus groups with students–one with primary education students and one secondary education and transition to adult life students–. On the one hand, results show the characteristics of the Dialogic Literary Gatherings that allowed these students to participate and learn. On the other hand, several improvements have been observed. First, regarding instrumental learning, students increased their motivation for reading, and improved their communicative and reasoning abilities and in their reading proficiency. Second, regarding students' behavior, conflict has reduced, solidarity attitudes have increased, and they have acquired dialogic and argumentative habits. Finally, at the emotional level, they gained self-esteem and confidence and feel more empowered to make their voice heard.

## Introduction

Literacy is an imperative educational need since it is basic for appropriate personal and social development. It is a condition for educational equality and enhances individuals' opportunities in life in current society (Katims, 2000; Lonsdale and McCurry, 2004). For this reason, educational actions that guarantee effective learning of literacy for all students and reduce the achievement gap between groups of learners are required. Specifically, people with disabilities have special difficulties in mastering basic skills of literacy assumed in society (Morgan et al., 2011). Besides, children with disabilities usually have overall less learning opportunities and tend to learn at a slower rate (Downing and Eichinger, 2003), which also compromises their learning of literacy. Therefore, fostering quality educational opportunities for these children focused on developing literacy skills is a pending challenge that would enhance the effectiveness and equity of educational systems.

Disability is understood to arise from the interaction between a person's health condition or impairment and the multitude of influencing factors in their environment (World Health Organization, 2020). According to this understanding of disability, limitations have to be brought into the social context (Grum, 2012), this is, there is a need of tackling the barriers of the context in order to achieve a greater social participation of individuals with disabilities and the subsequent reduction of their disabilities (Szmukler et al., 2014). In the field of education, it has implications for the overall development and learning of these children, and particularly for the learning of literacy. Since literacy is shaped through experiences and literacy practices in communities of practice (Barton and Hamilton, 2005), it is necessary to explore and identify practices and communities involved in literacy that maximize the participation and achievement of all learners, including those with disabilities.

Interactions are a crucial component of the social context that mediates learning and can create or overcome barriers to participation and learning. The work of Vygotsky (1979) revealed that learning and development occur first in the interactions between people and then it is interiorized at the individual level. Students learn when guided by an adult or when working with other more capable peers, by discussion, joint participation, encouragement, etc. Therefore, meaningful and positive interactions are needed to foster children's learning and development. This evidence is especially relevant in children with disabilities because of their intrinsic limitations for learning. Indeed, Vygotsky (1993) emphasized that educational approaches have to focus on children's strength instead of focusing on their disability. This is, on how the learning context and interactions can build on children's strengths. Students with learning disabilities who learn in inclusive environments in which interaction is enhanced, observing their peers' habits and behaviors as a model for their own (Lamport et al., 2012) achieve greater learning (Rea et al., 2002; Cosier et al., 2013; Kirby, 2017). Specifically, children with intellectual disabilities educated in inclusive settings tend to make more progress in literacy skills than those placed in special education settings (Dessemontet et al., 2012). According to research (Chitiyo et al., 2011) positive interactions and support, such as the ones that can be found in inclusive learning contexts, may explain increases in academic achievement of these children.

In this regard, research has shown that infants' social environment and interactions affect brain organization and functioning (Meltzoff and Kuhl, 2016; Dawson and Guare, 2018). The environment in which a person lives, as well as the actions of that person within that environment, play a role in plasticity, this is, in the ability of the brain to reorganize itself by forming new connections between neurons. Plasticity occurs, for example, in case of injury to compensate lost functions and, in general, whenever something new is learned and memorized (Grum, 2012). Plasticity is especially high in early and middle childhood, when children are more sensitive to developmental as well as environmentally driven changes (Buttelmann and Karbach, 2017). Thus, this ability of the brain for developing compensatory strategies is crucial for children with disabilities in their everyday life functioning. Regarding literacy, there is evidence that an environment rich in reading related events help developing some areas of brain (Kuhl, 2011; Hutton et al., 2015, 2020), thus contributing to brain plasticity that can help compensating difficulties that face people with disabilities.

In special schools, the learning possibilities derived from interactions with other children without disabilities are reduced, so these children cannot act as behavior models. Moreover, limitations inherent to the children and their classmates, who use to have cognition, communication and social skills affected (Szumski et al., 2017), reduce learning repertoire skills and strategies that can be exposed and shared in the class. Apart from that, these schools tend to implement more individualized work between the adult and the children, and this minimizes the opportunities for diverse learning interactions and peer learning. According to research, many behavioral traits are consequence of social interaction, which in the context of special schools can reinforce children's difficulties (Cantor and Kihlstrom, 2017). On the contrary, being surrounded by positive social incentives and inclusive educational settings helps develop a positive reorganization of higher mental functions (Grum, 2012). Therefore, it is especially important to identify venues to increase as much as possible learning interactions within special schools. This would approach these students' learning opportunities to those that their peers without special needs find in mainstream inclusive schools.

Literacy has also a crucial role in communication and language development. Literacy and communicative and reasoning abilities are all part of the instrumental learning contents that are considered necessary to enhance children's future educational and social opportunities (Light et al., 2008). Fostering communicative skills among children with disabilities is imperative to cope with everyday challenges throughout lifespan development, and appropriate dialogue and interaction opportunities in school foster these abilities by enhancing critical thinking and reasoning. Research has demonstrated that a dialogic-based interactional environment improves both communicative skills and language acquisition (Howes et al., 2008; Purcell-Gates et al., 2011). Therefore, it is important to promote dialogue and communication in literacy learning to improve children's literacy, communicative and argumentative abilities.

Research has already identified evidence-based educational actions that rely on quality interactions around learning to offer all students an optimal education. Successful Educational Actions (SEAs) were identified by the European research INCLUD-ED (Flecha, 2015), and have led to improvements in schools and communities across cultural and national boundaries (Garcóa-Carrión et al., 2017). Specifically, these actions have been transferred into special education settings achieving broader learning opportunities (Duque et al., 2020). Within these actions, Dialogic Literary Gatherings (DLGs) are an interactive dialogic-based learning environment where participants share and discuss the reading of classic works of universal literature, based on the principles of dialogic learning, which promotes freedom, respect toward diversity, and overcoming inequalities (Flecha, 2000). In DLGs interactions are based on egalitarian dialogue and oriented to the construction of collective knowledge (Soler, 2015) regarding the content of the reading and the topics that emerge from the discussion, which enables the development of critical consciousness. Therefore, DLGs focus on the development of literacy skills while reasoning and communicative abilities are practiced.

The scientific community has studied the efficacy of DLGs in various contexts and with diverse populations, showing positive results in different domains. Initially, the DLGs were identified as a tool to achieve optimal academic and social results in the literacy process in adult education (Flecha, 2000). Subsequently, the impact of the dialogic interactions facilitated by the DLGs has been replicated in other contexts including schools at different educational levels, from early childhood to secondary education (Flecha, 2015). Positive impacts have been observed in reading and language skills development (López de Aguileta, 2019), vocabulary acquisition (Hargreaves and García-Carrión, 2016) and prosocial behavior (Villardón-Gallego et al., 2018), all of them necessary abilities for appropriate academic and social development. Research has also shown that DLGs are a unique opportunity for students with disabilities to enhance their literacy achievement, motivation, and support to engage in a shared activity of dialogical discussion with non-disabled peers, increasing their opportunities to benefit from learning interactions, which tend to be scarcer for these students (Molina, 2015). This evidence suggests the possibility to transfer this intervention to other students with disabilities who are educated in mainstream or in special schools. There is already evidence that the implementation of interactive learning environments in special schools improves students' learning and behavior in this type of schools (Duque et al., 2020). Still, there is not yet research focused on the impact of DLG in special schools. This paper aims to fill this gap and analyse specifically the interactive learning environment created when DLGs are implemented in special schools, and how the learning interactions created around the reading and debate of classical books contribute to enhancing students' literacy and other potential benefits.

## Methods

An exploratory case study was conducted in a public special school which was one of the first experiences in implementing DLGs in a special school. Despite being a segregated learning context, even because of being placed distant from the urban centre, this school has been committed to offering quality and inclusive learning opportunities for these students. With this aim, professionals in the school implement Dialogic Literary Gatherings and other SEAs. At the data collection moment, they had been implementing DLGs between 2 and 3 years, which allowed evidence of DLGs impact be registered.

This case study was conducted in the framework of the broader research project *INTER-ACT. Interactive Learning Environments for the Inclusion of students with and without disabilities: improving learning, development and relationships* (Ministerio de Ciencia, Innovación y Universidades, 2017), which has the objective to assess the impact of interactive learning environments (DLGs and Interactive Groups) in learning, development and relationships of students with disabilities and to examine the conditions that may increase this impact. Specifically, this exploratory case study was aimed at addressing the following research questions: (1) Which are the characteristics of the interactive learning environment that is created when DLGs are implemented in special schools? and (2) Which are the benefits in terms of learning and development, if any, for students with disabilities participating in this interactive learning environment?

This instrumental case study (Stake, 1995) was conducted in order to achieve a deep and detailed understanding of how the DLGs are implemented in the special school, focusing on its characteristics and the different strategies carried out, and which are the improvements observed among students in terms of literacy development and other related improvements. In consequence, this study would enable to identify the relevant aspects in implementing DLGs in the special school context in order to recreate this interactive learning environment in other schools and achieve similar improvements.

Dialogic Literary Gatherings (DLGs) are a Successful Educational Action (SEA) in which participants, following principles of dialogic learning, share their ideas about classic works of universal literature (Flecha, 2000). They differ from normal reading training since they allow everyone's participation in an interactive environment, where all the interventions are equally valued. Moreover, incorporating works from universal literature maintains high expectations and provide culturally relevant resources and vocabulary.

The school implemented DLGs in primary and secondary education and in the transition to adult life course. With the aim of analyzing how were DLGs implemented across educational stages in the school, data collection was focused on the following groups:

1. The primary education group was composed of 20 students from 6 to 12 years old, all of them jointly participating in DLGs. In this stage students were affected by disabilities such as moderate intellectual disability, cerebral palsy, and autism spectrum disorder. Generally, the communicative and cognitive level of this group was much lower, so they need more scaffolding in order to participate. Some of them, in addition, presented behavioral or attention disorders.
2. The secondary education group contained 10 students aged between 12 and 16 with conditions including intellectual disability, autism spectrum disorder or behavior disorders. This group has also some communicative impairments. They have been 3 years implementing DLG once a week, with some families who attended to some of these sessions.
3. The transition to adult life course was composed of seven students aged between 16 and 21 with an intellectual disability. Their curricular level was equivalent to the 1st to 4th year of primary education (6–9 years old). They participate in DLG together with another transition to adult life course class, so they were finally about 15 participants in these sessions. Within this group there is more variability in terms of cognitive and communicative levels. They have been 2 years implementing DLGs once a week.

Qualitative data collection techniques were used with a communicative orientation (Puigvert et al., 2012). The data collection techniques used were in depth interviews and focus groups. These interviews and focus groups encompass different issues such as: strategies that facilitate implementing DLGs with children with disabilities, the characteristics of the implementation, results that emerged, etc. and were structured previously. Data was gathered from teachers of three different educational levels comprised in the school: primary education, secondary education and the transition to adult life course. This strategy allows to analyse teachers' different perspectives since they have different experiences and professional careers and encompass the overall educational intervention and impacts achieved. Specifically, two semi-structured interviews were conducted (to the school principal and a teacher of the transition to adult life course) and four focus groups, one with two secondary education teachers, one with three primary education teachers, one with two secondary education and a transition to adult life course students, and one with four primary education students. In the case of the focus groups with students, the conversation was always facilitated by a teacher familiar with the students to facilitate their expression and overall communication with them. Table 1 summarizes the information on the data collection techniques implemented.

**Table 1:** Data collection techniques.

**Technique**	**Profile**	**Group**	**DLG implementation**
Interview	School principal (female)	-	-
Interview	One Teacher (female)	Transition to adulthood course	2 years once a week
Focus group	Three Teacher (females)	Primary education	2 years once a week
Focus group	Four Students (three females, one male)		
Focus group	Two Teachers (females)	Secondary education	3 years once a week
Focus group	Two Students (males)	Secondary education	3 years once a week
	One Student (male)	Transition to adulthood course	2 years once a week

To ensure the research process's ethical integrity full details of the study objectives and procedures were explained to the participants, teachers and families prior to the start of the study. They were informed about the anonymous and voluntary participation and the confidentiality of the data collected of all participants. Informed consents were signed by all the participants or legal guardians after being properly informed. Ethical requirements were addressed following the Ethics Review Procedure established by the European Commission (2013) for EU research. The study was fully approved by the Ethics Board of the Community of Researchers on Excellence for All (CREA).

After data collection, interviews and focus groups were transcribed verbatim and subsequently analyzed. Drawing from the research questions, two main categories of analysis were stablished, which referred respectively to the strategies that were used in DLGs implementation and the improvements shown among participants. Within these two main categories, subcategories were created inductively based on the specific themes that emerged from the data. All names have been changed to pseudonyms to ensure the anonymity of the participants.

## Results

As follows, results are divided in two sections, firstly the characteristics of the implementation of DLGs in the special school are explained and, secondly, the improvements observed in several domains are addressed. In order to respond to the research questions, and for the sake of clarity, results are structured into such sections, however it is important to note that both aspects are connected, as the characteristics identified show strategies used in the transference of DLGs into the special school and are these strategies which enabled the improvements achieved.

### Characteristics of the DLGs When They Are Implemented in the Context of Special School

Our results show several strategies professionals used to adapt DLGs to the characteristics and needs of students in the special school. Some of these strategies are especially relevant in specific age groups, while others were used across all levels within the school.

#### Material and Procedural Adaptations That Enable Every Student to Participate

Due to the participating students' characteristics, in some occasions, adaptations are needed for DLGs to be inclusive for everyone. These adaptations aim to tackle the barriers that students may encounter because of their disabilities. This way, all the students can participate and share their knowledge. Depending on the characteristics of each student, different adaptations are needed. To this end, it is necessary that teachers know the characteristics of each student and identify which adaptation is required in each case for this student to participate. Overall, adaptations are focused in a way that, in the face of difficulties, it is not considered that a student cannot participate, but way is sought to enable him/her to equally participate.

In some cases, they used material adaptations. For instance, pictograms were an effective way in the school to facilitate students' expression, as they could express their questions and answers with this visual support. These adaptations were especially useful in the transition to adult life course. Some students with a lower literacy level used tablets and the story adapted into pictograms to facilitate their communication.

-Some students do not have literacy skills. We translate or adapt the chapter with pictograms. They work with a summary based on pictograms. Then, they can follow the activity with this adaptation…—Transition to adulthood course teacher

In the DLGs, students have read books such as *Romeo and Juliet, The Odyssey, Platero and I, Arabian nights, The metamorphosis*, etc. in secondary education, and *The Jungle Book, Peter Pan, Pinocchio*, for example, in primary education. Classic books of literature have accessible to buy adapted versions to different ages, which are faithful to the original book and incorporate rich vocabulary. Teachers in the school select the adaptation version that fits better to the characteristics of their students. In secondary education teachers stated not making any extra adaptation, to preserve the published version and not to alter its quality. In contrast, in primary education, they started using additional adaptations in the books, and they progressively were reading less adapted books.

-We do buy the classics that are already adapted, which perhaps from the outside may seem to be of a lower level by age that our children have, but no, we do not make any extra adaptation, neither on the vocabulary, nor include additional illustrations…—Secondary education teacher

-We have been improving, because we chose books that were like that (adapted into pictograms), then with capitalized words, and now a quite standardized book that we could find.—Primary education teacher

Both the school principal and the primary education teachers reported other material adaptations that were instrumental in facilitating everyone's participation. For example, these consisted of laminating the book sheets and tying some children's book to avoid throwing or breaking it. Instead of letting these students out of the activity, teachers found a way to avoid this disruptive behavior and include them with their peers.

-For example, we had to laminate the books. Because they were thrown, sucked and broken in the gatherings—School principal

-For example, we put a string to the book and tie it to the leg of the chair and at least, if they threw it, they wouldn't throw it to anyone—Primary education teacher

In other cases, the strategies used consisted of adapting the procedure of the DLG, in order to help in the development of the gatherings or in the internalizing of knowledge emerged in the gatherings. These adaptations are carried out following dialogic principles and with the aim of enhancing interaction and dialogic learning taking into account students' characteristics. A useful strategy consisted of preparing individually the specific interventions they are going to share in the gathering. This preparation of the gathering is carried out in all learning levels, facilitates the conversation, and having a more fluent dialogue and less guided by an adult. For example, a primary teacher states:

*-On Tuesday, we choose of topic and, as Marta said, we go deeper into that topic. There are several topics. If children want to ask, we decide to structure that question to bring it to that discussion session*.

-But we don't do it in group. We do it so that on Thursday it is the discussion in the gathering, because otherwise we reveal everything that's going to be in the gathering, if we do it in group. We help them to reflect and go deeper into it, without the others knowing, and then, for instance, you say: “and who would you like to ask about that?” and we prepare the question: “S., do you like…?” We prepare the question, and we talk among us about what they are going to ask, and they prepare a conversation, a dialogue, which is what is most difficult for us (…) but what has been most difficult for us is the conversation between them, which is not so directed by the adult. This is the work we prepare on Tuesdays—Primary teachers

Other strategies are used differently depending on the characteristics of the group. For instance, procedural adaptations in the transition to adult life course included using more direct questions to facilitate students' participation in the gathering:

-In the lower level group, you need to make more direct or guided questions to focus the dialogue. Because sometimes they choose a phrase or a word, and maybe they don't remember why they chose it or so. So, you have to ask him why he has chosen it, if it is because of this, or if it is because of that- Transition to adulthood course teacher

In primary education, one strategy they used is doing a gathering rehearsal to get used to be seated and listening to their peers. Moreover, they used a reinforcement programme with specific children that needed because of their attentional difficulties. These children were incentivized with a positive reward when he/she behaved well. It is important to highlight the fact that these strategies have been used to introduce children in the dynamics of the DLG and have been removed as long as students were familiarized with the dynamic.

-We thought that, of course, they didn't have the experience of sitting in a circle, talking and listening, they couldn't conceive that, so we did it for a couple of weeks, on Mondays when we arrived at class we did like a but what we did was sharing the weekend, but being in a circle, and that consists of talking and listening, participating, asking, answering. (…)—Primary teacher

Additional individual supports have been also used in some cases. For some primary education students these additional supports included the families' help, that could read the book with the children, and made less necessary the material adaptation.

-And we came to the decision that we were going to take the book, that it was adapted, so that it could be used in the 5-year-old class or in first grade because we thought that we were giving them the possibility that those who could read in lowercase letters would have the possibility of reading it for themselves, but the others, to have also the possibility (…) that their parents read it to them and not to make that adaptation to pictograms—Primary education teacher

In the case of students with more serious limitations, such as severe behavioral problems, the school found an effective strategy incorporating a person who functions as a reference figure (behavioral model) near these children to help them regulate their behavior, as the school principal explained.

-As far as the disability is concerned, it is true that we have students who are seriously affected, right? But we have tried that at least if they could come to the gathering for a while or at least that they remain seated with the group.—School principal

Importantly, teachers create these adaptations having always in mind the principles of the dialogic learning on which the DLGs are based. Teachers explicitly work on the internalization and practice of these principles. To achieve this, they use strategies such as having them visually accessible both in pictograms or in the blackboard, reviewing them before the gatherings start or working them each by one. For instance, secondary education students commented on this issue:

-What we do in the gatherings is to remind the most important ideas. Like “equality,” “creation of meaning,” “transformation” (…) When we are going to start, we say all the dialogic principles. And we also repeat the rules, like raising our hand and all that. (…) We sit in a circle, and there is a moderator, who takes notes and remind us rules such as that we have to be silent, we have to respect people, or when one speaks, we don't have to laugh at one who is speaking.—Secondary education students

In primary education, the dialogic learning principles have been adapted into norms that have been made visually accessible for all and, while maintains the principles' original meaning. In this way, they are easier to remember during the gathering.

-And it also served to understand what the rules are, to have them super clear, they were all in pictograms. So, instead of saying “shut up” so as not to interrupt the discussion, we showed the pictogram to the child that was difficult for him to be in silence, to teach him so that he regulates himself.—Primary teachers

After the gatherings, teachers and students also use some strategies to strengthen the learning emerged in these sessions. In secondary education, teachers explained that the class looked for the vocabulary that emerged in the gatherings and they did not know in the dictionary, and then, they created a panel with these words for each chapter. In primary education, they jointly think about each one's behavior after carrying out the gatherings to reinforce positive behaviors:

-Then, when we are finishing, we go through them one by one and say, for example, Claudia has had a good behavior? And everyone says yes. They are the ones who value the behavior of the gathering of all the classmates.—Primary education teacher

All of these strategies are carried out to conduct the Dialogic Literary Gatherings in a way that makes them inclusive for everyone. At the same time the basic principles of the DLGs are maintained, and any adaptation is aimed at facilitating participation and reinforce learning.

#### Coordination Among Different Educational Agents Inside and Outside School

Part of the strategy implemented to develop DLGs in the special school consisted of the coordination among diverse educational agents, which was identified as necessary and effective for the proper implementation of the DLGs, due to the students' characteristics and needs. Usually, the DLGs entail that students read the chapter alone and prepare an idea to share, but for students in this school it was difficult to do it by themselves. On the one hand, coordination among school professionals and families was a useful strategy to enhance students' preparation and participation in the gathering.

Families supported the students to prepare the reading. After this preparation, they had the opportunity to make the most of the DLGs sessions and to dialogue about the idea or ideas they had previously thought (with or without help) to contribute to the gathering. Involving families was crucial not only because they have a relevant role for strengthening children's routines and learning habits, but also because students need to think and practice what they want to share. Despite the help that comes with the implication of families, in some cases it has been complicated to engage relatives. In these particular cases, teachers are the ones who could do this preparation work with these children previously to the session.

Students in all the school educational levels could benefit from this support to prepare their participation in the gathering. In primary education, children read the chapter during the weekends with their families, which help them to argue their ideas:

-On Friday they take the text home to do that shared reading with the families, and in their notebook, with their families, they take note of the chapter, the page, the line or a word and the idea of why, by arguing why they have chosen that word. All this is the work with the family—Primary education teacher

Families have also participated in the gatherings with their children in the school. According to the primary education teachers, parents' participation allowed to create a particular atmosphere of collaboration, gave them a different perspective, developed high expectations and promoted a more normalized behavior among the students.

-*The participation of the families, how they feel, how they see them, how they see the gathering when their families come. It becomes very special; a very special atmosphere is created. Last year we saw when the family is available and we opened them up to participate and that atmosphere was very beautiful, because you could see how their son or daughter was doing and the rest. (…). The intention is to continue inviting families to see this type of activity and participate because it also benefits self-esteem and feeling special.—Primary education teacher*

In secondary education and in the transition to adult life courses families' collaboration is also present. In secondary school, students counted with their family support with the reading, and in the transition to adult life course, coordination between families and teachers has been crucial to help teachers to understand children's ideas and helping to express them:

-*Teacher: Who helps you read? Who helps you read at home*.

-Student: My mother.—Secondary education student

-Parents also help us a lot, because sometimes they write in the diaries: “My son has chosen this word for this, for this and for this.” To see if it then matches the version he gives. Because at first, maybe with his mother or father will say that he has chosen that word because… for some special reason. And then in class, maybe he'll say another one or he doesn't remember.—Transition to adult life course teacher

Besides families, teamwork and adequate coordination among the school teachers were necessary for the successful implementation of the DLGs in the school, leading to better achievement of the educational goals. Teacher coordination was impregnated with the same egalitarian dialogue typical of the dialogic learning and the DLGs, thus educational objectives were established based on an egalitarian dialogue with every stakeholder, and shared purposes were agreed. In this sense, teachers of all educational levels mentioned that they always work together and jointly prepare the materials and discuss methodological adaptations. In this regard, they highlight as especially important their joint participation in evidence-based and dialogic teacher training in the form of pedagogical gatherings. As teachers explained, this allowed them consolidating the theoretical and empirical basis of their practice and being updated on successful educational methodologies that have proven to be effective.

-I believe that the first factor, the most important one is training (…) I think that coordination between teachers is very important. Training, coordination, preparation and high expectations. - School principal

#### Taking Advantage of Diversity as a Strategy in DLGs

Being immersed in an environment characterized by diversity has also been used as a relevant strategy in achieving improvements regarding the learning and development of children with disabilities in the special school. Although fewer evidence was found regarding this topic, it is relevant to highlight the school teachers' perception of the transformative potential of diversity. This diversity in the context of special schools included both taking advantage of the existing diversity and incorporating more diverse interactions in the DLGs dynamic. The more variety of characteristics, experiences and behaviors they interact and become familiar with, the more they can learn. In this vein, it was highlighted as important the opportunities students have to be in touch, interact and dialogue with other students and with adults, like students' families. They can bring different knowledge and experiences to learn from, in an interactive learning environment such as the DLGs. They can also act as role models who can induce more appropriate behavior among children.

-Being able to interact with other children and to be in contact with other children who use other expressions, or adults who participate in the gatherings, who use other expressions in the gatherings which children assume little by little.—Secondary teachers

-Obviously we had to put more people of reference, models of appropriate behavior, and then the same in terms of groups, we try to start incorporating students from other classrooms that we know are complicated and difficult into a class that we see that works.…—School principal

### Benefits of Student Participation in DLG in the Special School Context

The improvements observed in students due to their involvement in Dialogic Literary Gatherings in the special school are diverse. These improvements include instrumental learning, and particularly literacy abilities and communicative skills, which are the learning contents more directly addressed with the DLG. However, other improvements related to students' behavior, empowerment, and selfesteem have been found, which show the comprehensive approach of the DLGs.

#### Benefits in Terms of Literacy and Communicative Abilities

One of the first impacts of participating in DLGs is the increase in the motivation for reading. Teachers and students across educational levels agree that this motivation observed emerge from the opportunity of shared reading provided by the DLGs. The contents of the debates and the participative and egalitarian basis of the gatherings foster children's motivation and joy for reading. This opposes with the previous experience of the teachers of the secondary education classes. They mentioned that before implementing DLGs it was usual for students to refuse reading and, after starting to participate in DLGs, they live reading differently, they enjoy it. This transformation in their motivation resulted in a more joyful attitude and transformed their predisposition to improving their reading abilities. In this regard, students who started participating with little reading abilities, became motivated to learn to read alone the chapter for the gathering and participate in it.

-Now they do not reject reading in the same way. They already experience it in another way, they can enjoy more than a reading.—Secondary education teacher

The motivation for reading is also shown by children's desire to join in the gatherings. Both teachers and students were aware of this increase in motivation. As primary teachers observed:

-And they're even thinking about what they're going to share. “I wanted to say that,” which for the students is very positive, it's a moment they're looking forward to, to being the two classes together, which normally they're not (…). Super positive—Primary education teacher

One of the participating students, explained it in this way:

-Well, there was a classmate who was not interested in the gatherings. But then, after a few days we started to read together, all in a group, that person started to like the gatherings. But people used to say that the gatherings were silly, that they were worthless. Anyway, they said these things. But then, after a few days, people liked them more. And, for instance, there are people who help me, and I help the others too. We help each other, for instance some of us read better than others. People are doing quite well with the gatherings—Secondary education student

Significantly, this increased motivation for reading is related with the increase in reading proficiency facilitated with Dialogic Literary Gatherings. As the school principal stated:

-As for the improvement of the reading I see it clearly—School principal

As motivation for reading is enhanced in interactive learning situations, the opportunities to learn from these interactions also increase and, indeed, entailed an improvement in reading skills. Improving reading competence through reading motivation is crucial for internalizing such learning and extending it over time and other contexts. This improvement is constantly mentioned along the interviews and focus groups at the different educational levels.

In this sense, secondary and primary education teachers reported some specific cases of students who did have a significant improvement, especially in reading, as a result of participating in the DLGs:

-In the case of a student who had serious behavior problems, who had left all the academic work aside because of the behavioral problems, when we took the group and this student joined it, we resumed academic work. At first, he did not remember anything, not even writing. He was a child who did write, but it was illegible. And in two courses, it's true, his progress has been, was extraordinary. Because he took the chapter with him, he read it, and understood as the others—Secondary teacher

-Yes, let's see Julio, until this year he was reading in capital letters, he had been working on the transition to lowercase letters and it was very difficult for him and that is something that he is working on and he reads much better in lowercase. (…) And in the case of Pedro, he has advanced a lot in reading, until last year when he began with capital letters, this year you can notice the progression in his reading, in lowercase as well.—Primary education teachers

DLG have also been observed to help children enhance their communicative abilities. They could elaborate longer phrases, acquire new vocabulary from the book and elaborate more structured discourse, as primary teachers stated:

-I think there has been an improvement in the coherence of the dialogue, in which a topic is being talked about, I think that everyone understands the topic that is being talked about in a certain way, they are talking about that topic, in that sense the attention has improved, that would be positive. The structuring of sentences, everyone is very clear that they will spontaneously say just a word, but then they try to structure, and it is all because of the habit that we are working on of sentence structuring. These small changes are the ones that are observed in each session—Primary education teacher

Even in the cases when there was a low level of expressive language development, clear improvements were observed in the ability to structure an idea, as the school principal showed with the example of one of the students:

Before he only said “blue” now he is able to say “my favorite color is blue because I see it in the sky” —School principal

In other cases, when language proficiency was not only mediated by a disability but also because children came from another country with a different language, teachers also described improvements in language acquisition as a result of participating in DLGs:

-Kevin recently arrived, he is French, therefore it has been impressive in Kevin the benefit that the gatherings have brought him in terms of oral expression because, of course, he spoke in French, well he speaks in French and you can see that he makes more appropriate structures, but of course, because he has the imitation, the students learn by imitation and he has the imitation of the others. —Primary education teacher

-This child who arrived here and didn't speak any Spanish, he speaks French and suddenly, well in the first gatherings we made very direct questions, but in the fourth one, I remember that I wrote it down because I was the moderator that day and we were talking and he contributed something, it was a word, but it was what we were talking about and it was in a language that 2 months ago was unknown to him. —Primary education teacher

These language improvements can be related to a combination of factors, according to the characteristics of the DLGs and the evidence collected. First, the high quality of books they read, which are humanity's great literary creations and provide a rich language input. Second, participants have to think and elaborate an intervention to share with the group, which entails an additional cognitive effort. Third, the opportunity to listen to peers and teachers' interventions, who act like behavior models, facilitate them to learn new speech abilities and argumentative skills. Finally, as they have not only to understand the reading but also to link its content, which often reflects socially relevant issues, with their lives, it entails making connections, comparisons, elaborate arguments, and explain them. These improvements were observed both in primary and secondary education students:

-It has also helped… well I don't know if it's only me, but I suppose for many people gatherings have also been helped to reflect on many things in his life. For example, many of the gatherings have helped me to think. Because I used to think in a different way. – Secondary education student

-I also see them sharing certain things, certain topics of daily life in our lives and in their lives, which many times are very similar. And the sharing of everyday issues is reflected in everyone. For example, we were talking about a little dog, and one said: “Ah well I also have a dog” and the other one already wants to know the name of his friend's dog and when he is going to walk it. So, yes, in sharing everyday topics—Primary education teacher

#### Increase of Students' Prosocial Behavior

Apart from the improvements in terms of reading and language abilities, other improvements referred to students' behavior and their relationship with others. Results show that by implementing DLGs students learn to respect opinions and argue their posture and have dialogues around it. This has an impact on the coexistence and the prosocial behavior of these children.

Teachers observed the development of prosocial behavior in terms of greater solidarity, empathy and tolerance among students. For example, in secondary education, DLGs facilitated an increased acceptance of diversity in different forms, including religion and life beliefs:

-We have seen differences between cultures, there are Muslim and Roma girls, and this interaction and acceptation with the diversity, even of religion, life beliefs, we see it.—School principal

Another example of the improvement in empathy is highlighted by primary teachers, that comment the following:

-In the gatherings, new proposals emerge, this is, we are talking and Emilio, who is in a wheelchair, tells us that he wants to go down the slide, and a question comes out: Emilio, do you want to play soccer tomorrow? (…) if we have talked that Emilio has not been able to play, the next day it comes out: Let's go for Emilio! They all go to play soccer with him.—Primary education teacher

In addition, children became more able to express whether they agree or not with someone else and explain why, with arguments, in a dialogic and respectful environment. In this regard, students have developed a greater introspection ability to identify and admit in their own inappropriate behaviors. This ability is developed in the gatherings because of the respectful and dialogical environment that is created, which provides a context in which no one judges or evaluates the others, and participants' empathy and acceptation emerged. This was specially observed in secondary education; both teachers and students reflected on it:

-They had not had a space where they could express themselves freely, and where they would be heard and not judged for what they were saying. And they have learned that too. A… “we can have different opinions, it's okay, you can argue, I can argue, and we can have a dialogue.” So, I think the gathering has created that. That space that they didn't have until now.—Secondary education teacher

-Well, I've seen that I've seen myself alone many times because of that. I've also had to think, I've had to say: “Well, I'm doing this wrong,” I'm doing… you know, right? As a result of the gatherings, I thought about what I was doing wrong. And finally I could know what it was, that it was very unfair, and many other things. Secondary education student

Apart from internalizing these habits, students were able to generalize these prosocial habits and attitudes to other contexts, such as family or community. This transferability is a relevant outcome since communicating and providing arguments is key to getting along in society. Both primary and secondary education teachers observed this improvement.

-This is giving them the possibility, when they go out on the street, when they are in the parks, to be able to ask, to make some contribution.—Primary education teacher

#### Empowerment and Enhanced Self-Esteem

Finally, an impact at the emotional level was observed, which is something extremely important to students with special educational needs and disabilities. In the interviews, issues such as gaining selfesteem and empowerment emerged. Teachers from all educational levels greatly appreciated the improvement they have perceived in students' self-image and self-esteem. Being engaged in respectful and meaningful interactions, children with a low confidence level have built a stronger identity. This occurs with many students who arrived at the school having given up, because of the treatment received in other schools. However, by participating in the DLGs, they have recovered high expectations on their capabilities. To achieve this self-confidence, a climate of trust and knowing that no one would laugh at them is of capital importance, and they found it in the DLGs. Teachers in primary and secondary education and in the transition to adult life course described how they observed this improvement among their students:

-We value very positively the self-concept that children have created for themselves. (…) We have seen that they have more confidence in themselves, that they value themselves more, that they think they can have friends, and they have friends all of a sudden, right? Well, they are 16-year-old and it is the first time they go out on the weekend their schoolmates, that had never happened before.—Secondary education teacher

-That self-esteem, to feel more secure, for example in Nestor, I have not seen him, but they have told me that many times he was paralyzed, closed… And now I see him participating, with self-esteem, feeling secure…—Primary teachers

-In this group there is a student who before this year had problems of adaptation, problems of being misunderstood, that nobody understood him… his family even considered leaving the school. This year he started with a new group, new classmates, with the dynamics of the gatherings, the interactive groups, the brave club and so on. He saw a space where he had the floor. A space in which he could express what was going on in his head. The problem he had was a very low self-esteem. Very low, very low. So, to have the opportunity to express himself, to feel supported by classmates… He knows that no one will laugh at what he says. That has given him security. A climate of trust has been created in the classroom. (…) Little by little, oral expression began to flow. He began to tell and relate the chapter to some experience. He began to participate in all the gatherings. Not only at the school but also at home they noticed improvement. Because now he told them more things. He was more open. He increased his self-esteem.—Transition to adult life course teacher

This enhancement of self-esteem not only contributed to students' wellbeing but being more confident helped students learn to make their voice heard in gatherings itself and in other contexts where they want to give their opinion. DLGs create a respectful space where every intervention is valued. This environment enhances confidence to ask or give their opinion, an ability required in other fields of life.

-Thanks to the gatherings they have been given a voice in other spaces, (…) in the assembly of student representatives, in the lunch time assembly which I have attended, where they have wanted to ask for improvements and where they did it. (…) And in playground, that is, “hey I need to talk to you,” “I respect my turn if I'm talking to someone else.” We were very used to deciding about them. So, when that breaks down and the dialogue is egalitarian, they get very empowered—School principal

## Conclusions

After analyzing students' and teachers' voices, characteristics, strategies, and improvements were identified related to the implementation of Dialogic Literary Gatherings with students in the special school context. This evidence opens a new field of study regarding the possibilities of implementation of DLG. The results also show that in a context where interactions with typically developing peers is limited, it is crucial to significantly enhance social interactions in special school to improve the education of students with disabilities. This is in line with Vygotsky (1979) ideas about the relevant role of dialogue and interactions in children's development with and without disabilities and the evidence that promoting social interactions impacts cognitive (Howes et al., 2008) and language (Purcell-Gates et al., 2011) development, fundamental in literacy learning.

With regard to strategies and characteristics related to DLGs identified by the teachers and students involved, some common issues emerged. First, turning children's limitations into possibilities introducing adaptations in different ways is essential to enable everyone's participation.

Transforming the barriers that can appear when working with children with disabilities is a crucial aspect in order to achieve a positive impact on these students' education. This transformative approach was observed in the adaptations carried out in order to enable everyone to participate. Adaptations for particular students are not usually made not to altering whole-group strategy, being lack of training and school support possible causes for no adapting, according to research (Scott et al., 1998). This case study shows how enabling, by different ways of adaptations, students with disabilities to participate, all of them have enough resources to join in the activity, while the whole group activity is maintained.

Second, the relevant role of the families' support and teachers' coordination based high expectations of students, and on the implementation of the dialogic principles to professional teamwork. Family is a decisive factor in children's education, and in the context of special education families take an important role in students' development. Involving families in dialogic reading and learning, improves students' literacy skills (Huebner and Payne, 2010) and also improve literacy communication behaviors of all family members (Brannon and Dauksas, 2012). Regarding teachers and expectations, previous research has demonstrated that teachers holding high expectations of students' level of achieving positively affects student motivation and engagement (McKown and Weinstein, 2008; National Research Council, 2004 cited by Rubie-Davies et al., 2006; Murray and Pianta, 2007). Our study shows that high expectations can be built in the context of special education, overcoming deficit-based perspectives, and this occurs when educational interventions are based on promoting learning interactions with teachers, classmates and families.

The implementation of DLG following these strategies has led to some improvements, which are in line with the results of previous research that has showed how Dialogic Literary Gatherings enhance academic (Flecha, 2000), social (Alvarez et al., 2018; García-Carrión et al., 2020), and emotional (Racionero-Plaza, 2015) outcomes in different contexts and cultures (Aubert, 2015). On the one hand, benefits in terms of students' literacy were identified. Specifically, motivation for reading and reading proficiency was enhanced, as well as communicative and argumentative skills. Previous studies showed that DLGs bring improvements in reading skills, for example, by improving vocabulary acquisition (López de Aguileta, 2019). DLGs also increase students' motivation (Aubert, 2015; Hargreaves and García-Carrión, 2016) which also has a potential impact on reading skills, since motivation for reading influences daily reading which results in increased reading achievement (Sonnenschein and Munsterman, 2002; Brannon and Dauksas, 2012). Specific cases of students with disabilities showing an improvement in motivation for participating in the gatherings and learning to read have been reported (Molina, 2015). The case study reported here shows that improvement in motivation and learning in DLG occur also in the context of a special school, where enhancing literacy skills is an important challenge. By improving reading skills, which is essential to succeed academically (Goldman, 2012), DLGs contribute to a quality education that enhances their academic learning and, consequently, improve their life opportunities (Smith et al., 2017; Gil-Lacruz et al., 2020).

In terms of communicative and argumentative habits, the study results highlight the increase in argumentative skills. Scientific literature highlights that students talking about what they have read and receive feedback in a dialogical way regarding their ideas is a mechanism for promoting language learning (Valdez-Menchaca and Whitehurst, 1992). Our results also show students' ability to link interpretations of literary books they read in the DLG with their lives. This impact of DLG was also identified in other studies, which showed outcomes related to argumentative and literacy skills (López de Aguileta, 2019). This is a relevant issue, since improvements in oral expression help reduce the impact of disability (Molina, 2015). In addition, it is known that children, specifically those with disabilities, need to be involved in learning experiences that make sense for them when literacy is being worked (Basil and Reyes, 2003), because they should perceive it as legitimate (Mertens, 2012). This is in line with the principle of creation of meaning in dialogic learning, in which DLGs are based (Garcia et al., 2018) and that is manifested when they see reflected contents of the readings in their own experiences.

On the other hand, an increase in students' prosocial behavior was observed, involving solidarity and tolerance attitudes and dialogic habits. This fact is also in line with other studies, which have demonstrated DLGs to improve relationships and kindness interactions (García-Carrión et al., 2020).

This particular way of learning, based on interaction and dialogue, also has shown to promote children's prosocial behavior (Villardón-Gallego et al., 2018). In particular, solidarity and tolerance feelings have emerged through DLGs in these students. Participants also internalized dialogic habits which improved coexistence, such as respecting and arguing different opinions. This is, they learned how to provide arguments on their posture, and how to do it based on respect. Since their interpretations have to be based on claims instead of on power positions (Oliver and Gatt, 2010), no student's interpretation was more valid than anyone else's and this enhanced a respectful group climate. These results show that the communicative and argumentative habits that were learnt served at the same time to enhance the learning in the language and literacy domain and to improve the classroom climate and peer relationships, showing the comprehensiveness of this dialogic interactive environment.

Finally, children gained self-esteem and empowerment. DLGs have demonstrated gains in selfesteem and empowerment among participants in diverse contexts (Aubert, 2015; García et al., 2017).

Self-esteem is viewed as an evaluative judgment reflecting the individual's sense of self-worth (Cosden et al., 1999), so it has a strong connection with empowerment. In this case, this improvement is especially relevant since children with special educational needs usually have negative self-perception (Kloomok and Cosden, 1994; Alesi et al., 2012). Nevertheless, peer relationships are associated with higher self-esteem in children with disabilities (Renick and Harter, 1989; Kloomok and Cosden, 1994). Thus, maintaining quality interactions between students ends in an increment of self-esteem. DLGs achieve this gain in security and empowerment by generating good interactions between children and creating meaning (Aubert, 2015).

However, this case study presents some limitations. One of the limitations is inherent in being a single case study, such as having data from only one school or not having a control group to compare its impact. This research is an exploratory study that analyzes a specific educational practice among children with disabilities, so sample chosen was by convenience and no representative. Nonetheless, it demonstrates that it is possible to implement DLGs, based on dialogue and argumentation about classic works of literature, in a challenging context as is a special school. Based on this evidence more special schools can start implementing this practice, and new research could extend the analysis on the potential improvements achieved in these schools. In this regard, this research allowed identifying areas of improvement, which could be further analyzed. Finally, outcomes presented could have been biased because of the nature of the qualitative data collection techniques. In this sense, carrying out only qualitative techniques could led to social desirability bias, as well as less concrete results. Nevertheless, the aim of this research is not to compare it with others but to provide qualitative elements of practice for others to replicate. Further research using quantitative data and standardized quantitative instruments could provide more accurate evidence on the magnitude of these improvements. We argue that more research is necessary to analyse the impact of DLGs on students with disabilities both in the special education context and in inclusive environments, and enhance their transferability to new schools, to improve the educational experience and achievement as these students deserve.

## Data Availability Statement

The raw data supporting the conclusions of this article will be made available by the authors, without undue reservation.

## Ethics Statement

The studies involving human participants were reviewed and approved by Ethics Board of the Community of Researchers on Excellence for All (CREA). Written informed consent to participate in this study was provided by the participants' legal guardian/next of kin.

## Author Contributions

RV-C conceptualized the research. AF-V did the literature review and drafted the article. RV-C, PM, and IT reviewed and edited the manuscript. All authors have read and agreed to the submitted version of the manuscript.

## Conflict of Interest

The authors declare that the research was conducted in the absence of any commercial or financial relationships that could be construed as a potential conflict of interest.
